# Developing a Water Quality Index (WQI) for an Irrigation Dam

**DOI:** 10.3390/ijerph14050439

**Published:** 2017-04-29

**Authors:** Celia De La Mora-Orozco, Hugo Flores-Lopez, Hector Rubio-Arias, Alvaro Chavez-Duran, Jesus Ochoa-Rivero

**Affiliations:** 1 National Research Institute for Forestry, Agriculture and Animal Production, Km 8 Tepatitlan-Lagos de Moreno, Jalisco, 47600 Tepatitlan de Morelos, Jalisco, Mexico; flores.hugo@inifap.gob.mx (H.F.-L.); chavez.alvaro@inifap.gob.mx (A.C.-D.); 2 Faculty of Animal Science and Ecology of the Autonomous University of Chihuahua, Periferico Francisco R. Almada, km 1, Carretera Chihuahua-Cuauhtémoc, Colonia Zootecnia, 31031 Chihuahua, Mexico; rubioa1105@hotmail.com; 3 Experimental Center La Campana, National Research Institute on Forestry, Agriculture and Animal Production, Km 33.3 Carretera Chihuahua-Ojinaga, Aldama, 32910 Chihuahua, Mexico; ochoa.jesus@inifap.gob.mx

**Keywords:** water quality index, irrigation, Jalisco, Mexico

## Abstract

Pollution levels have been increasing in water ecosystems worldwide. A water quality index (WQI) is an available tool to approximate the quality of water and facilitate the work of decision-makers by grouping and analyzing numerous parameters with a single numerical classification system. The objective of this study was to develop a WQI for a dam used for irrigation of about 5000 ha of agricultural land. The dam, La Vega, is located in Teuchitlan, Jalisco, Mexico. Seven sites were selected for water sampling and samples were collected in March, June, July, September, and December 2014 in an initial effort to develop a WQI for the dam. The WQI methodology, which was recommended by the Mexican National Water Commission (CNA), was used. The parameters employed to calculate the WQI were pH, electrical conductivity (EC), dissolved oxygen (DO), total dissolved solids (TDS), total hardness (TH), alkalinity (Alk), total phosphorous (TP), Cl^−^, NO_3_, SO_4_, Ca, Mg, K, B, As, Cu, and Zn. No significant differences in WQI values were found among the seven sampling sites along the dam. However, seasonal differences in WQI were noted. In March and June, water quality was categorized as poor. By July and September, water quality was classified as medium to good. Quality then decreased, and by December water quality was classified as medium to poor. In conclusion, water treatment must be applied before waters from La Vega dam reservoir can be used for irrigation or other purposes. It is recommended that the water quality at La Vega dam is continually monitored for several years in order to confirm the findings of this short-term study.

## 1. Introduction

It is important to assess the health of water bodies to develop strategies to improve water resource and watershed management. Most ecosystems around the planet are experiencing problems associated with water scarcity and quality. In other words, water resources are at risk [1]. Natural processes and anthropogenic activities influence water quality, causing degradation in surface and groundwater and affecting their potential use for human and animal consumption, agriculture, recreation, industry, and others [2]. The chemical compounds in water bodies like dams are strongly influenced by water use in the watershed and by hydrological and biological cycles [3]. As well, the main components in natural waters are ions dissolved in rainwater, soil and rocks in catchments [4]. Ultimately, water quality in a dam is the result of complex interactions among numerous factors such as rainwater, groundwater, evaporation, and human activities throughout the catchment. For example, during the rainy season there is significant inflow of numerous compounds. Nevertheless, dilution resulting rainwater can reduce the negative impact on water bodies [5]. Several authors have reported the effect of land use on surface and groundwater quality [6,7], noting that changes in land use can impact directly water quality parameters. 

Physical and chemical parameters can be used to evaluate the water quality and are directly associated with water use. However, studying these parameters separately does not clearly define water quality. In any case, the parameters must meet pre-established standards for water use in a particular region or country; otherwise, treatment before use will be required if the water does not meet the standards [8]. A good alternative is to integrate a set of physical and chemical variables to develop a water quality index (WQI), in which a large number of water parameters result in a single number that represents the level of water quality [9,10]. The final index includes the values obtained from direct measurement of substances and physical and chemical variables obtained through analyses of water samples [11,12]. The WQI is clearly a useful tool to evaluate water quality given a criterion for surface water classification [13,14,15,16]. It has been well documented that many surface water bodies in Mexico have pollution problems [17,18,19,20], including the La Vega dam. In addition to irrigation, the dam reservoir is a source for fishing, which is an important activity for about 100 families in the area. Several studies have found problems of pollution of dam waters related to the sugar cane industry, domestic wastewater, and agricultural runoff [21]. The objectives of this study were to develop a WQI for La Vega dam and to analyze possible temporal changes in water quality. 

## 2. Materials and Methods

**Figure 1:**
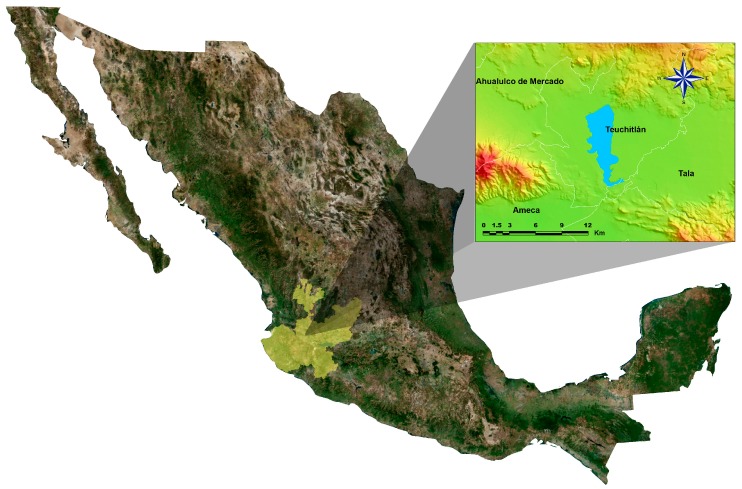
Location of La Vega Dam in Jalisco, Mexico.

The study was carried out at La Vega dam located in the municipality of Teuchitlan in the state of Jalisco, Mexico (approximately 1300 m a.s.l., at 20°33′50″ N and 20°47′40″ N and 103°47′30″ W and 103°51′20″ W) (Figure 1). The dam was built from 1952 to 1956 for irrigation purposes. Its capacity is about 44 million m^3^, with an area of 1950 ha [22] and is considered the largest water body in Western Central Jalisco. This aquatic ecosystem meets some of the water requirements of the towns of Ameca, San Martín Hidalgo, Cocula, Tala, Teuchitlan, Ahualulco del Mercado, Villa Corona, Zapopan, el Arenal, Tequila, Magdalena, San Juanito de Escobedo, Etzatlan, Tecolotlan, Atemajac de Brizuela, and Zacoalco de Torres.

Water samples were collected at seven randomly selected sites around the dam in March, June, July, September, and December 2014 to determine the water quality dynamic over the course of a year. The months selected for water sampling include the dry (March, June, and December) and rainy season (July and September) in the area. In addition, March, June, and September are when most irrigation takes place in the area. A total of 35 samples were collected. The sites were selected at random after dividing the entire area of the dam reservoir into 1 km^2^ quadrats using Google Earth^TM^ software (Google, Mountain View, CA, USA). Sites were located with GPS (Etrex Brand, Garmin, Olathe, KS, USA). The water samples were collected in polyethylene bottles according to Mexican standards [23]. The collected water samples were properly stored (4 °C) and transported to the laboratory for further analysis. The following 18 parameters (Table 1) were quantified in the laboratory: potential hydrogen (pH), electrical conductivity (EC), dissolved oxygen (DO), total dissolved solids (TDS), total hardness (as CaCO_3_), alkalinity (CaCO_3_), NO_3_, SO_4_, TP, Cl^−^, Ca, Mg, Na, K, B, As, Cu, and Zn. The parameters used for the WQI were selected for their importance for irrigation and regional crops. 

The pH level, temperature, EC, DO, and TDS were measured in situ with a portable device (Hanna, Woonsocket, RI, USA) according to the Standard Methods for the Examination of Water and Wastewater [27]. Atomic absorption and spectrophotometry were used to quantify the remaining parameters. 

The WQI was established in three steps the first of which was to normalize individual values used to design the index. This was done to establish a correspondence of the results obtained for each parameter with a variable scale of 0 to 100 based on the maximum values established in official standards. A value of 100% indicates optimal natural conditions, while 50% indicates significant constraints in water use. Table 2 and Table 3 present the parameters used to calculate the WQI with their corresponding normalizations [34]. The second step in determining the WQI was to assign numerical weights to the parameters, which were established according to their importance in normal criteria of quality. A team of persons specialized in water quality determined the weight of the parameters. It should be noted that the assigned weights had been applied in a previous works on Chapala Lake [35]. Table 4 shows the assigned weights.

The third step was calculating the WQI by applying the following Equation [35]:

$$\mathrm{WQI}=\frac{\sum Wi\cdot Pi}{W\cdot Pi}$$

where *Wi* is the parameter value (%), and *Pi* is the weight given to each parameter.

The results were interpreted according to the intervals, where 4 of the categories of the WQI are as follows: 0 to 39% indicates highly contaminated water; 40–59% poor water quality, 60–90% good quality, and 90–100% excellent quality [35]. 

## 3. Results and Discussion

### 3.1. Characteristics Water in the La Vega Dam Reservoir

Table 5 shows the mean concentrations and the standard deviations of 18 parameters obtained at seven sampling sites in 2014. The pH concentrations were constant throughout the study period, with values ranging from 8.17 to 8.61. Concentrations of pH in the range of 7.2 to 8.3 irrigation waters can cause problems mainly associated with the availability of nutrients (P, Fe, Cu, Mg, and Zn). EC concentrations were around 400 dS·m^−1^ at all sites. According to international standards include restrictions on EC concentration in irrigation waters. In the specific case of sugar cane, the limits that affect the potential yield are as follows: no restriction when EC is below 1.7 dS·m^−1^, light to moderate restriction when EC is around 1.7–19 dS·m^−1^, and maximum restriction when EC is over 19 dS·m^−1^. Therefore, the results of our study indicate no restriction on the use of water from La Vega dam for irrigation purposes.

DO concentrations were in the range of 5.34 to 6.18 mg·L^−1^. Some authors [36] have stated that DO concentrations in water under 5 mg·L^−1^ can cause oxygen deficiency in plant roots, with serious consequences for agricultural production. The TDS was in the range of 219 to 236 mg·L^−1^ throughout the study, with slight differences observed among the seven sampling sites. These levels were below international TDS limit of 500 mg·L^−1^ for the safe use of water for irrigation. Hardness presented some variation, with values ranging from 31.7 to 61.2 mg·L^−1^. Chloride concentrations varied, with values in the range of 30.1 to 36.4 mg·L^−1^, which according to Mexican and international standards do not represent any risk for use in irrigation. Table 6 shows that NO_3_ concentrations were higher at Sites 3 and 6 (1.37 mg·L^−1^) than at Site 4 (0.59 mg·L^−1^). Some authors [37] have reported negative effects on sensitive crops of using irrigation water with nitrogen concentrations above 5 mg·L^−1^. However, most crops do not experience negative effects with nitrogen concentration below 30 mg·L^−1^ [37]. Crop sensitivity varies according to the growing stage. Nevertheless, high nitrogen concentrations can benefit crop growth [37]. Concentrations of TP were generally low and in the range of 0.15 to 0.59 mg/L. Mexican standards do not provide any specific limits for P in irrigation water. However, maximum concentrations of 0.025 mg·L^−1^ in lakes and dam reservoirs are desirable.

SO_4_ concentrations varied throughout the study, ranging from 9.80 to 14.1 mg·L^−1^. Crops like sugar cane require higher sulfate concentrations (94 kg·ha^−1^) than other crops (corn: 47 kg·ha^−1^, rice: 20 kg·ha^−1^) [38]. 

Chemical elements such as B, Ca, Mg, K, and Na, alone or in combination with others, are toxic to the environment and are often present in water bodies. Heavy metals such as As, Cu, and Zn can negatively affect water quality, as well as soil quality when they are present in irrigation water. Some of these elements are important for plants growth, but in high concentrations they can be harmful. High concentrations of nitrates and heavy metals can be harmful to humans, and high concentrations of P can result in eutrophication in water bodies [39,40]. Ca and Mg concentrations ranged from 5.09 to 7.25 mg·L^−1^ and from 5.57 to 6.20 mg·L^−1^, respectively. Na was high (85.3 mg·L^−1^) at Sampling Site 3 and lower (69.6 mg·L^−1^) at Site 4, while the highest concentration was observed near the Salado River. The concentration of K was constant from 11.3 to 12.4 mg·L^−1^, while mean values of B concentrations were from 3.75 to 4.24 mg·L^−1^. 

According to Mexican standards [41], the maximum permissible level of B in water for irrigation purposes is 0.75 mg·L^−1^ for what are termed “sensitive” crops and 3 mg·L^−1^ for less sensitive crops. It was evident that B concentrations in the water of La Vega dam B exceed the limits for even tolerant crops. It has been reported that the amount of B absorbed in soil and sediment depends on pH concentration and B concentration in the solution. Absorption is generally highest when pH is between 7.5 and 9.0 [39]. The water from La Vega dam has B levels that are toxic for crops. With pH values above 8, the risk of absorption increases, limiting the use of the dam water for irrigation. 

Mean As concentrations ranged from 0.13 to 0.18 ppm, which exceed Mexican limits for waters of varying uses [41]. The use of water with high levels of As for irrigation can inhibit plant growth and reduce yields [42]. In addition, As in soil can react with elements such as Ca, Mg, and Fe [43]. Table 5 also shows that Cu and Zn concentrations were constant. 

### 3.2. Water Quality Index

There were no significant differences in WQI values among the seven sampling sites along the dam over the study year (Table 6). While only light fluctuations were observed among sites, seasonal variations were observed. Table 6 shows that WQI values fluctuated from poor, to medium and good. For example, WQI values were below 60 units for all sampling sites in March and June, except at Sampling Site 6 where WQI was 60.8 in June. According to the classifications for the different water uses, values below 60 indicate that the water requires treatment before use for irrigation or other purposes. The low WQI values in March concur with findings of others authors [1], who observed that water quality decreased in spring in Northwestern Spain. Water quality improved slightly in July and September, with WQI values between 62 and 73. We hypothesize that this improvement is the result of the rainy season in this particular year of study (2014). However, we do not have the environmental data to confirm this. Nevertheless, it is recommended that more data be obtained over several years in order to confirm the effect of environmental conditions on water quality at the La Vega dam. It should be noted that several authors have reported the polluting effect of rain and climatic conditions on the dynamics and water quality of rivers and reservoirs [44,45,46,47]. However, rainy season runoff can introduce pollutants such as nitrogen and phosphorous, which contribute to the blooming of water hyacinths. The WQI decreases again in December after the rainy season, presenting values ranging from 56 to 66 units, which represents a classification of poor to medium. Water treatment is recommended to make dam waters more fit for irrigation or other purposes. 

The lowest WQI values were observed in the southern part of the reservoir where the Salado River drains into the reservoir, while the highest water quality values were found in the northern part of the reservoir. Water quality in La Vega dam is notably affected by anthropogenic activities such as agricultural production, the sugar cane industry, and urban growth. In a valuation of the Luis L. Leon dam in Aldama, Chihuahua, Mexico [48], the researchers suggested that anthropogenic activities in the area have been responsible for changes in water quality in the reservoir. 

Other researchers [49] calculated a WQI to evaluate historic water quality of Lake Dokan in the Kurdistan Region, Iraq. They observed that water quality had degraded from excellent to good and then to poor from 1978 to 2009, mainly due to human activities and population increases near the reservoir. The authors of [50] designed a WQI for Lake Mallathahalli in India and concluded that water quality in the lake had decreased to a classification of poor by 2012. Nevertheless, water from Lake Mallathahalli is still considered suitable for irrigation according to Indian standards. 

From the data set obtained in this research, we have an approximation of the water quality at La Vega dam in 2014. This research also identified natural contamination of high concentrations of B and As carried by the Salado River as one of the main sources of pollutants in La Vega dam. The source of the Salado River is La Primavera forest, a volcanic area with high B and As content [51,52,53]. This research identified that parameters such as B, As, and Na have the most significant effect on reducing the WQI. 

## 4. Conclusions

From this first water quality index determined for La Vega dam using data collected over one year (2014), several pollutants were identified that affect water quality in the reservoir. The study verified that high concentrations of B, Na, and As enter the reservoir with the inflow of the Salado River in the southeastern part of the dam. The northern part of the dam has been identified as the main source of nutrients such as P and N, originating from domestic wastewater transported by the Teuchitlan River. In general, the concentrations of the analyzed parameters are affected by a number of factors, such as the rainy season, effluents from the sugar industry, and agricultural runoff. These finding can support planners and decision-makers in developing strategies for the sustainability of La Vega dam and the economic activities that take place in the surrounding area. Current research is focused on intensive monitoring of the water at La Vega dam and its inflows, and some remediation strategies focus on the waters of the Salado River before they reach the dam. It is highly recommended to collect data over several years to identify the water quality dynamics at La Vega dam and to confirm the effect of several environmental conditions. This research provides the groundwork for continuing monitoring of the reservoir. 

## Figures and Tables

**Table 1:** The evaluated parameters, the relevant Mexican standards, and the analytical method used.

Parameter	Mexican Standards	Analytical Method
pH	NMX-AA-008-SCFI-2000 [24]	Potentiometer
EC (dS·m^−1^)	NMX-AA-093-SCFI-2000 [25]	Potentiometer
DO (mg·L^−1^)	NMX-AA-012-SCFI-2001 [26]	Potentiometer
TDS (mg·L^−1^)	Standard Methods 2540 c [27]	Potentiometer
TH (mg·L^−1^)	NMX-AA-072-SCFI-2001 [28]	Titration
Alk (mg·L^−1^)	NMX-AA-072-SCFI-2001 [28]	Titration
Cl^−^ (mg·L^−1^)	NMX-AA-073-SCFI-2001 [29]	Potentiometer with silver nitrate
NO_3_ (mg·L^−1^)	NMX-AA-082-1996 [30]	Cadmium column (Griess-Ilosvay Met.)
SO_4_ (mg·L^−1^)	NMX-AA-074-1981 [31]	Spectrophotometry
TP (mg·L^−1^)	NMX-AA-029-SCFI-2001 [32]	Spectrophotometry
Ca (mg·L^−1^)	NMX-AA-51-SCFI-2001 [33]	Atomic Absorption
Mg (mg·L^−1^)	NMX-AA-51-SCFI-2001 [33]	Atomic Absorption
Na (mg·L^−1^)	NMX-AA-51-SCFI-2001 [33]	Atomic Absorption
K (mg·L^−1^)	NMX-AA-51-SCFI-2001 [33]	Atomic Absorption
B (mg·L^−1^)	NMX-AA-51-SCFI-2001 [33]	Spectrophotometry
As (mg·L^−1^)	NMX-AA-51-SCFI-2001 [33]	Atomic Absorption
Cu (mg·L^−1^)	NMX-AA-51-SCFI-2001 [33]	Atomic Absorption
Zn (mg·L^−1^)	NMX-AA-51-SCFI-2001 [33]	Atomic Absorption

Note: All Mexican Standards may be consulted as open access information. EC: Electrical conductivity; DO: Dissolved oxygen; TDS: total dissolved solids; TH: Total hardness; Alk: Alkalinity; TP: Total phosphorous.

**Table 2:** Normalization of individual values.

Parameter	pH	EC	DO	TDS	TH	Alk	Cl^−^	NO_3_	SO_4_	Value
		dS·m^−1^	mg·L^−1^	mg·L^−1^	mg·L^−1^	mg·L^−1^	mg·L^−1^	mg·L^−1^	mg·L^−1^	%
Analytical value	1/14	>16.00	0	>1.500	>1.500	>1.500	>400	>55	>250	0
	2/13	12	1	1.5	1	1	350	50	225	10
	3/12	8	2	1	800	800	300	45	200	20
	4/11	5	3	800	600	600	250	40	175	30
	5/10	3	3.5	600	500	500	200	35	150	40
	6/9.5	2.5	4	500	400	400	150	30	130	50
	6.5	2	5	400	300	300	100	25	100	60
	9	1.5	6	300	200	200	50	20	75	70
	8.5	1.25	6.5	200	100	100	25	10	50	80
	8	1	7	100	50	50	10	5	25	90
	7	<750	7.5	<100	<25	<25	<10	<5	<10	100

**Table 3:** Normalization of individual values.

Parameter	TP	Ca	Mg	Na	K	B	As	Cu	Zn	Value
	mg·L^−1^	mg·L^−1^	mg·L^−1^	mg·L^−1^	mg·L^−1^	mg·L^−1^	mg·L^−1^	mg·L^−1^	mg·L^−1^	%
Analytical value	>0.65	>250	>50	>90	>35	>3.0	>0.30	>0.30	>4.0	0
	0.6	225	45	80	30	2.5	0.2	0.28	3.6	10
	0.55	200	40	70	25	2	0.18	0.26	3.2	20
	0.5	175	35	60	20	1.5	0.14	0.24	2.8	30
	0.45	150	30	50	15	1	0.12	0.22	2.4	40
	0.4	120	24	40	10	0.7	0.1	0.2	2	50
	0.3	100	20	30	8	0.5	0.09	0.15	1.6	60
	0.25	75	15	20	6	0.4	0.08	0.1	1.2	70
	0.2	50	10	10	4	0.3	0.07	0.05	0.8	80
	0.15	25	5	5	2	0.2	0.06	0.03	0.4	90
	<0.10	<20	<5	<5	<1	<0.1	<0.05	<0.01	<0.02	100

**Table 4:** Weight given to the parameters in calculating the water quality index (WQI).

Parameter	Weight (*Wi*)	Parameter	Weight (*Wi*)
pH	2	TP	1
EC	3	Ca	2
DO	1	Mg	3
TDS	2	Na	5
TH	2	K	5
Alk	3	B	5
Cl^−^	3	As	4
NO_3_	1	Cu	2
SO_4_	5	Zn	2

**Table 5:** Mean and standard deviations of the parameters in water from the seven sampling sites.

	Sampling Site 1	Sampling Site 2	Sampling Site 3	Sampling Site 4	Sampling Site 5	Sampling Site 6	Sampling Site 7
pH	8.23 ± 0.56	8.27 ± 0.50	8.17 ± 0.56	8.42 ± 0.43	8.61 ± 0.66	8.43 ± 0.42	8.41 ± 0.56
EC (dS·m^−1^)	449 ± 165	447 ± 144	483 ± 149	457 ± 138	480 ± 184	445 ± 136	447 ± 123
DO (mg·L^−1^)	5.34 ± 0.83	5.39 ± 1.41	5.42 ± 0.75	5.47 ± 0.61	6.01 ± 0.55	6.18 ± 0.47	6.14 ± 0.50
TDS (mg·L^−1^)	219 ± 80.1	219 ± 71.3	237 ± 73.7	224 ± 67.8	236 ± 72.9	218 ± 67.2	219 ± 60.7
TH (mg·L^−1^)	45.2 ± 38.7	61.2 ± 44.3	59.2 ± 43.8	42.5 ± 39.5	57.7 ± 46.8	31.7 ± 40.9	42.9 ± 40.6
Alk (mg·L^−1^)	14.2 ± 12.1	13.3 ± 12.5	14.8 ± 11.3	13.1 ± 12.4	18.1 ± 14.7	9.96 ± 12.8	11.3 ± 13.6
Cl^−^ (mg·L^−1^)	31.9 ± 11.5	36.4 ± 8.14	37.9 ± 10.2	36.2 ± 7.03	37.3 ± 6.96	30.1 ± 10.2	32.3 ± 9.97
NO_3_ (mg·L^−1^)	0.76 ± 1.01	0.54 ± 0.57	1.37 ± 2.24	0.50 ± 0.62	1.08 ± 2.01	1.37 ± 1.84	0.96 ± 1.27
TP (mg·L^−1^)	0.59 ± 0.32	0.45 ± 0.15	0.45 ± 0.24	0.30 ± 0.19	0.15 ± 0.12	0.31 ± 0.18	0.29 ± 0.13
SO_4_ (mg·L^−1^)	10.3 ± 7.52	11.1 ± 9.39	11.1 ± 6.12	14.1 ± 9.22	9.80 ± 2.22	11.7 ± 14.1	13.8 ± 7.24
Ca (mg·L^−1^)	7.25 ± 6.20	6.99 ± 6.29	6.80 ± 6.15	6.81 ± 6.34	9.26 ± 7.51	5.09 ± 6.57	6.14 ± 6.72
Mg (mg·L^−1^)	5.47 ± 2.07	5.39 ± 2.05	5.40 ± 1.80	5.86 ± 1.58	5.93 ± 2.48	6.20 ± 1.66	5.57 ± 1.87
Na (mg·L^−1^)	76.5 ± 28.5	77.2 ± 26.7	85.3 ± 25.3	69.6 ± 37.8	79.3 ± 25.3	74.6 ± 23.9	76.0 ± 19.9
K (mg·L^−1^)	11.6 ± 2.83	11.9 ± 1.81	12.2 ± 1.86	11.9 ± 1.53	11.9 ± 2.10	12.4 ± 1.60	11.3 ± 1.38
B (mg·L^−1^)	3.80 ± 0.84	3.85 ± 0.86	4.24 ± 0.73	3.84 ± 0.61	3.89 ± 0.62	3.80 ± 0.59	3.75 ± 0.59
As (mg·L^−1^)	0.17 ± 0.02	0.16 ± 0.03	0.18 ± 0.04	0.15 ± 0.01	0.16 ± 0.01	0.13 ± 0.02	0.13 ± 0.02
Cu (mg·L^−1^)	<0.01	<0.01	<0.01	<0.01	<0.01	<0.01	<0.01
Zn (mg·L^−1^)	<0.01	<0.01	<0.01	<0.01	<0.01	<0.01	<0.01

**Table 6:** WQI values in water from the La Vega dam reservoir in five months and at seven sampling sites.

Date	Sampling Site 1	Sampling Site 2	Sampling Site 3	Sampling Site 4	Sampling Site 5	Sampling Site 6	Sampling Site 7
March	57.34	56.60	54.15	56.81	56.17	59.04	58.51
June	55.32	54.79	54.89	55.85	55.53	60.85	58.72
July	63.72	62.98	62.77	64.89	64.68	66.38	67.66
September	70.96	69.26	65.85	73.30	69.26	69.79	69.57
December	61.17	63.11	60.85	61.70	56.06	61.17	66.70
